# A Systematic Review of Aflatoxin B1 Contamination in Livestock Feed and Detection Methods in Ethiopia

**DOI:** 10.1002/vms3.70405

**Published:** 2025-05-13

**Authors:** Sisay Weldegebriel Zeweld, Enquebaher Kassaye Tarekegn, Meressa Abraha Welearegay

**Affiliations:** ^1^ Department of Veterinary Public Health and Food Safety Mekelle University College of Veterinary Sciences Mekelle Ethiopia; ^2^ Department of Chemistry Mekelle University College of Natural and Computational Sciences Mekelle Ethiopia

**Keywords:** aflatoxin B1, contamination, detection methods, Ethiopia, livestock feed, systematic review

## Abstract

Aflatoxin B1 (AFB1) contamination in Ethiopian livestock feed poses significant risks to both animal and human health due to poor hygiene and inadequate storage practices. This study focuses on specific cereal grains, such as maize and groundnuts, which are commonly used in livestock feed but have been underexplored in terms of their role in AFB1 contamination, especially under local farming and storage conditions. A literature search was conducted from June 2024 to November 2024, utilizing databases like Google Scholar and African Journals Online. Articles were screened on the basis of titles and abstracts, with duplicates removed using EndNote. Only English‐language publications from 2000 onwards were included. Quality assessment adhered to Centre for Reviews and Dissemination (CRD) and Cochrane guidelines, and statistical analysis was performed using Statistical Package for the Social Sciences (SPSS). Non‐English studies were excluded to focus on accessible research for Ethiopian stakeholders, ensuring the relevance of findings. Out of 96 retrieved works, 79 remained after eliminating duplicates and pre‐2000 studies. After screening, 30 full‐text articles were assessed, resulting in 16 exclusions. Ultimately, 14 studies, primarily cross‐sectional, qualified for review. AFB1 contamination levels varied widely, from 2.4% to 100%, across different regions in Ethiopia. This study underscores the urgent need for robust monitoring and interventions to combat AFB1 contamination in Ethiopian livestock feed. Analysis of covariance (ANCOVA) analysis revealed significant differences in contamination levels based on detection methods, highlighting the need for standardized approaches to ensure consistency in reported findings. This study underscores the urgent need for robust monitoring and interventions to combat AFB1 contamination in Ethiopian livestock feed. Standardized detection methods and aligning Ethiopia's regulations with international standards like Codex Alimentarius are essential for safeguarding public and animal health.

## Graphical abstract

**Article Highlights**:

**•**Examined AFB1 contamination in Ethiopian livestock feed from 2000 to 2024;

**•**Identified significant contamination levels ranging from 2.4% to 100%;

**•**Analysed detection methods, highlighting variability in effectiveness;

**•**Recommended improved monitoring and standardizing detection practices;

**•**Emphasized region‐specific interventions for more effective control;

These highlights emphasize the novel results and key findings from our research, aiding discoverability and providing a clear overview of the contributions of the study.

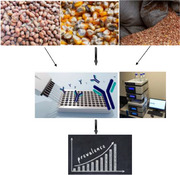

## 1 Introduction

### 1.1 Background

The prevalence of aflatoxin contamination in livestock feed in Ethiopia and the methods employed for aflatoxin detection have been extensively studied. Aflatoxins, particularly aflatoxin B1 (AFB1), AFB2, AFG1, and AFG2, are the main mycotoxins produced by *Aspergillus flavus*, *Aspergillus parasiticus*, and other species, including *Aspergillus nomius*, *Aspergillus tamarii*, *Aspergillus pseudotamarii*, *Aspergillus bombycis* and *Aspergillus australis*. These mycotoxins pose significant health risks to both animals and humans due to their toxic and carcinogenic effects (Alameri et al. 2023; Tadele et al. 2023). Studies have reported AFB1 levels as high as 213 µg/kg in Addis Ababa dairy feed, significantly exceeding the permissible limit of the European Union (EU), which is 5 µg/kg for dairy animal feed, indicating its potential to spread between humans and animals through the food chain (Negash 2018). Contamination is a pressing food safety concern in dairying activities, as naturally occurring microorganisms spoil animal feed at the farm level and during storage, leading to significant animal health issues (Setsetse 2019).

Sources of contamination include environmental pollution, industrial effluents, faecal matter, insects and endogenous toxins introduced during harvesting, transportation, storage and processing (Tadele et al. 2023; Kemboi et al. 2020). Dairy animal feed is particularly prone to contamination by aflatoxigenic fungi and their toxins. This risk extends to dairy products like milk, where aflatoxin M1 has been detected in raw milk samples in Ethiopia (Dinkissa and Hailu 2022; Tefera et al. 2022). The US Food and Drug Administration (FDA) strongly recommends that animal feed be appropriately manufactured and labelled to avoid health risks (Negash 2018).

Globally, aflatoxin regulations emphasize maximum permissible limits to ensure food and feed safety. For instance, the EU has set maximum tolerable limits for AFB1 in feed at ≤5 µg/kg for dairy animals and ≤20 µg/kg for other livestock. Similarly, the Codex Alimentarius Commission recommends strict aflatoxin limits, emphasizing a farm‐to‐fork approach in controlling contamination. Sub‐Saharan Africa, including Ethiopia, faces unique challenges in adopting these standards due to limited infrastructure, inconsistent policy enforcement and lack of awareness among farmers (Negash 2018; Garcia‐Alvarez‐Coque et al. 2020; Sangvikar 2021). These challenges underline the need for context‐specific approaches that bridge the gap between local realities and international best practices. This review aims to address these disparities by identifying actionable insights tailored to the agricultural systems of Ethiopia.

In developing countries like Ethiopia, dairy cattle feed commonly includes forage (e.g., hay, wheat bran and wheat middling), cut‐and‐carry pasture, home‐grown cereals, commercial concentrates and Atella, a by‐product of home‐brewed beer (Desta 2023; Ebrahimi 2020). Due to limited crop availability for human consumption, Atella is increasingly replacing commercial concentrates, despite its potential contamination risks (Zewdie et al. 2011). These feed types, particularly Atella, hay, cut‐and‐carry pastures and limited commercial concentrates, are highly susceptible to aflatoxin contamination during pre‐harvest, post‐harvest and storage stages (Tadesse et al. 2020; Shiferaw et al. 2022; Kitigwa 2023). The reliance on locally sourced, often inadequately stored feed creates a unique contamination landscape distinct from that of developed countries, necessitating context‐specific interventions.

Livestock feed serves as a nutritional source for animals raised for meat, milk, eggs, wool, or labour (Ndudzo et al. 2023). In Ethiopia, common feed mixtures consist of maize, sorghum, wheat by‐products, barley by‐products and peanut or groundnut by‐products, all of which are vulnerable to AFB1 contamination at various stages of harvest, processing and storage (Karangiya et al. 2016; McCuistion et al. 2019). Poor hygiene in handling equipment and feeding practices further exacerbates aflatoxigenic fungal contamination (Getabalew et al. 2019). Economic pressures also compel dairy farmers to stockpile feed during harvesting seasons, yet prolonged storage under unsuitable temperature and humidity conditions promotes mould growth and aflatoxin production (Getabalew et al. 2019; Motbaynor et al. 2021).

Although substantial research in Ethiopia has examined AFB1 contamination in livestock feed, studies rarely investigate specific cereal grains, such as maize, sorghum and groundnuts, as distinct contributors to contamination (Gizachew et al. 2016; Mesfin et al. 2018; Mengesha et al. 2023). Detection methods employed in Ethiopia include conventional microbiology, enzyme‐linked immunosorbent assay (ELISA), polymerase chain reaction (PCR) and high‐performance liquid chromatography (HPLC). Although these methods are widely used, they vary in terms of sensitivity, specificity and cost, which can impact their adoption at the local level (Tefera et al. 2022; Mahato et al. 2019; Yilma et al. 2019). The lack of widespread access to advanced technologies like HPLC and PCR poses significant barriers to consistent and reliable aflatoxin monitoring in Ethiopia. By comparing these methods to global practices, this review seeks to demonstrate cost‐effective and scalable solutions for low‐resource settings. Incorporating advanced detection methods and aligning aflatoxin regulations of Ethiopia with international standards could significantly improve feed safety. For example, promoting good agricultural practices (GAP) and implementing hazard analysis and critical control points (HACCP) systems, as practised in developed nations, could help mitigate risks throughout the feed supply chain (Negash 2018). This review builds on these insights by emphasizing the practical steps needed to close the implementation gap, offering recommendations tailored to the socio‐economic and agricultural realities of Ethiopia.

Therefore, this systematic review aims to provide insights into the prevalence of AFB1 contamination in Ethiopian livestock feed and evaluate the methods used for detection. Understanding these aspects is vital for implementing effective interventions, updating regulatory frameworks and improving feed safety standards in Ethiopia.

### 1.2 Rationale

AFB1 contamination in Ethiopian livestock feed presents a significant challenge to food safety and animal health. Although prior studies have examined overall contamination levels, gaps remain in understanding the role of specific cereal grains (e.g., maize, sorghum and groundnuts) as primary contributors to AFB1 prevalence. This systematic review aims to consolidate and analyse existing research, identifying key risk factors and evaluating detection methods to inform targeted interventions. A crucial aspect of this review is the comparative analysis of detection techniques, ranging from conventional methods like thin layer chromatography (TLC) to advanced approaches such as HPLC. Understanding the strengths, limitations and applicability of these methods in Ethiopia's resource‐limited settings will provide actionable insights for improving aflatoxin monitoring. Additionally, Ethiopia's regulatory framework for aflatoxin control remains misaligned with international standards such as Codex Alimentarius. This review will explore context‐specific strategies to enhance compliance, strengthen monitoring systems and support evidence‐based policymaking for better aflatoxin management. By synthesizing existing literature, this study seeks to bridge knowledge gaps, recommend practical solutions and guide stakeholders in adopting effective aflatoxin control measures, ultimately safeguarding public and animal health in Ethiopia.

### 1.3 Research Questions

- What is the prevalence of AFB1 in livestock feed in Ethiopia according to existing research?
- How diverse are the methods employed for AFB1 detection in Ethiopian livestock feed?

These research questions aim to evaluate the extent of AFB1 contamination in Ethiopian livestock feed by synthesizing findings from existing studies. Furthermore, they explore the diversity of detection methods, including conventional and advanced technologies, to understand their application and effectiveness in the Ethiopian context.

## 2 Methodology

### 2.1 Data Sources and Search Strategy

A thorough literature search was conducted from June 2024 to November 2024, focusing on five major databases subscribed to by Mekelle University and/or available through Open Access. These databases included Google Scholar (http://scholar.google.com), African Journals Online (AJOL) (http://www.ajol.info), ScienceDirect (http://www.sciencedirect.com), Scopus (http://www.scopus.com), PubMed (https://pubmed.ncbi.nlm.nih.gov/) and JSTOR (http://www.jstor.org). A manual search of the reference lists of the retrieved articles was performed to ensure the inclusion of all relevant resources. The search used specific groups of keywords and their synonyms, connected by the ‘AND’ and ‘OR’ operators (Berrang‐Ford et al. 2015). The research questions were clearly defined and designed to identify articles or studies discussing the prevalence of AFB1 in livestock feed, the methods of detection and studies specifically related to Ethiopia, with a focus on systematic reviews. The four key search terms, based on the title ‘Systematic Review on the Presence of Aflatoxin B1 in Livestock Feed and Detection Methods in Ethiopia’, were (1) AFB1 prevalence in livestock feed in Ethiopia, (2) livestock feed contamination with AFB1 in Ethiopia, (3) detection methods for AFB1 in animal feed and (4) systematic review of AFB1 in Ethiopian livestock. These terms were combined using Boolean operators (AND, OR) to refine the search, for example, (‘Aflatoxin B1 prevalence’ OR ‘Livestock feed contamination’) AND (‘Detection methods’ OR ‘Aflatoxin B1 detection’) AND (‘Ethiopia’ OR ‘Ethiopian livestock’) AND (‘Systematic review’).

### 2.2 Study Selection and Data Extraction

An electronic search was conducted to locate published literature in English. Although non‐English studies were excluded, this approach ensured the review focused on research accessible to Ethiopian policymakers and stakeholders, aligning with its practical application goals. The reference lists of relevant articles were manually examined. References from different databases were imported into EndNote Version X8 and scrutinized for duplication, with any duplicates removed. Titles and abstracts were initially screened to determine eligibility. If the abstract indicated that the study was pertinent to the topic, the full articles were obtained and thoroughly reviewed. The eligibility of articles was determined on the basis of whether they met the inclusion criteria. Data were extracted on important variables, including the location of the study area or region, type or species of study animal, livestock feed type (sample), sample size, method of detection, AFB1 levels detected (i.e., levels higher than the maximum tolerable limit set by the US FDA and the Ethiopian Standard Agency, which is >5 µg/kg), prevalence of AFB1 and publication year. For studies to be considered relevant for this review, they needed to report the prevalence of AFB1 (positive samples with an AFB1 level greater than the permissible limit of >5 µg/kg) in livestock or animals and describe the method of detection used in Ethiopia. In this systematic review, the mean AFB1 level was one of the variables of interest and was used in the analysis of the retrieved works.

### 2.3 Inclusion and Exclusion Criteria

Research articles written in English, with abstracts indicating relevance to the research topic, were included. Studies published since the year 2000 were also considered. Articles that did not meet the inclusion criteria based on their abstracts were removed, including those with duplicate references. Studies published before the year 2000 were excluded. Works that did not mention the prevalence of AFB1 in livestock or animals, or those that did not describe the method of detection in Ethiopia, were also excluded. Additionally, review articles or studies focusing solely on human aflatoxin exposure, as well as studies on AFB1 that did not include livestock feed, were excluded.

### 2.4 Quality Assessment

The guidelines of the Centre for Reviews and Dissemination (CRD) and the Cochrane Handbook for Systematic Reviews of Interventions were followed to ensure the quality of the systematic review. After clearly formulating the research question, an extensive literature search was conducted using major databases that cover aflatoxin and health‐related research. To prevent selection bias, the research question was explicitly defined, and thorough literature searches were performed across major databases, incorporating clear inclusion and exclusion criteria. A systematic selection process was used to screen the works, thereby enhancing the reliability and validity of the findings.

### 2.5 Evidence Synthesis and Statistical Analysis

In the process of evidence synthesis from the selected quantitative studies, a thorough summary of the extracted variables of interest was created. The Statistical Package for the Social Sciences (SPSS) version 20 was used to analyse the various variables extracted from the studies. Descriptive statistics were employed to summarize, organize and present the data in a way that clearly illustrates the variability and distribution of the extracted information. The analysis of covariance (ANCOVA) was applied to assess whether there were statistically significant variations in group means concerning the dependent variable (AFB1 levels), allowing for the evaluation of the influence of covariates and main effects. This analytical approach enabled a comprehensive and meaningful interpretation of the quantitative findings across the selected studies.

## 3 Results

### 3.1 Accessed Works

The search, focused on the ‘prevalence of aflatoxin B1 in livestock feed and detection methods in Ethiopia’, was conducted from October 2023 to January 2024 and resulted in 96 works. After removing duplicates and works published before 2000 G.C., 79 works remained.

### 3.2 Screening Procedures and Outcomes of Obtained Documents

**FIGURE 1:**
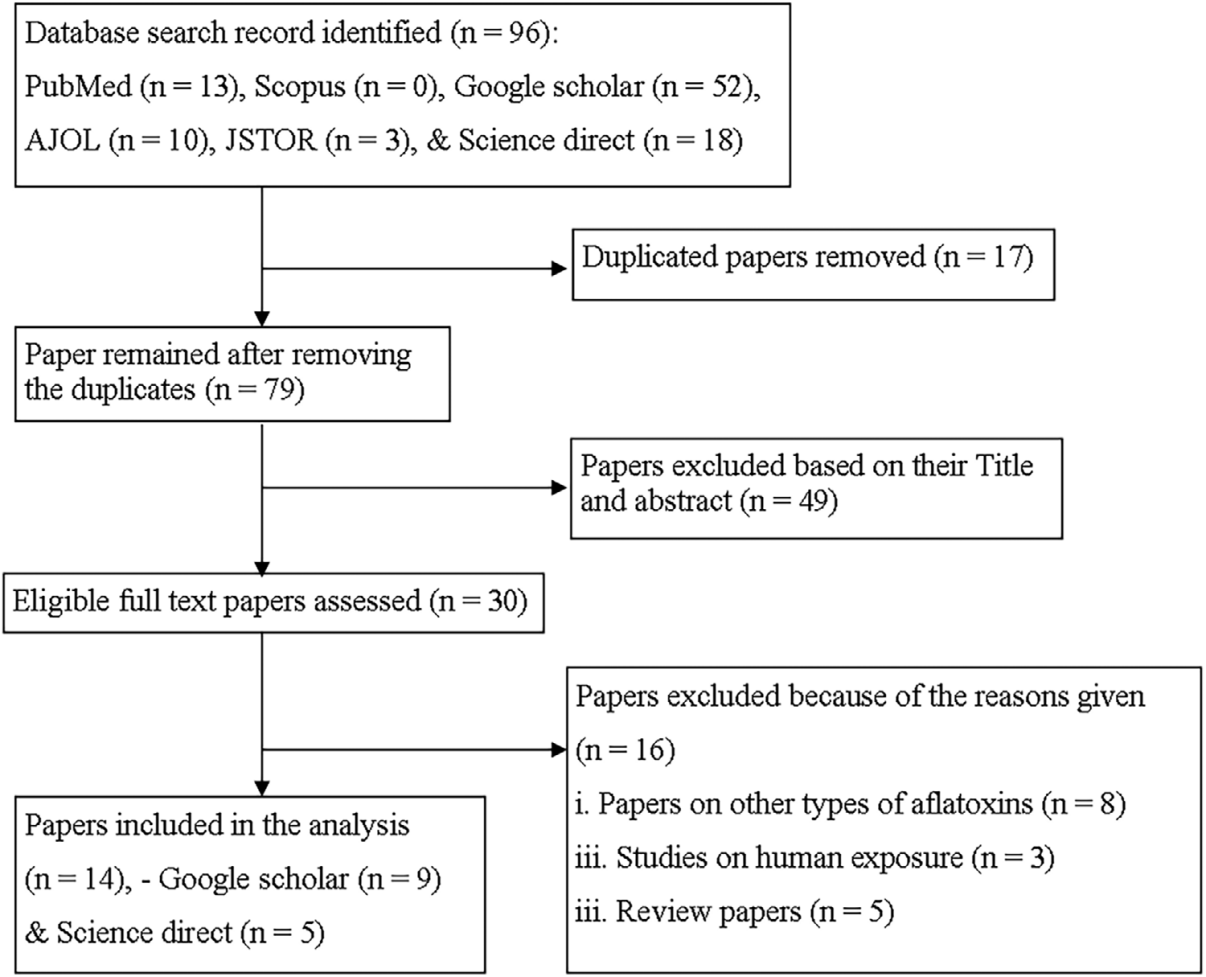
Flow chart for screening procedure of studies.

After removing duplicates and works published before 2000 G.C., 79 works were screened on the basis of their title and abstract, considering the inclusion and exclusion criteria. This process led to the exclusion of 49 works. The remaining 30 works were reviewed in detail, and 16 were excluded for various reasons. In the final stage, 14 works met the qualifications for the review process (Figure 1). All the research works that qualified for the review were conducted using a cross‐sectional study design.

In the initial search, the majority of the works were retrieved from the Google Scholar database (54.2%, 52/96), followed by ScienceDirect (18.75%, 18/96). However, after the final screening, 64.3% (9/14) and 35.7% (5/14) of the works included in the review were from the Google Scholar and ScienceDirect databases, respectively.

### 3.3 Spatial Distribution of the Retrieved Works

**FIGURE 2:**
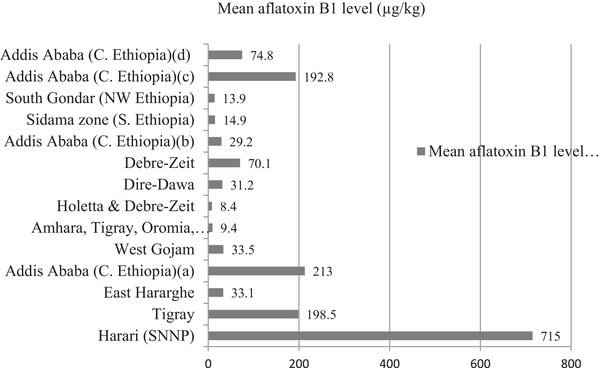
Regional distribution of mean aflatoxin B1 levels in livestock feed (µg/kg).

The spatial distribution of the retrieved works reveals a diverse range of study areas or regions within Ethiopia, each associated with specific prevalence rates of AFB1 contamination in livestock feed. The studies reported aflatoxin prevalence rates, ranging from 2.4% to 100%, emphasizing the diverse contamination levels in livestock feed. Urban areas, like Addis Ababa, and moderate contamination levels were observed in regions like West Gojam and Holetta/Debre Zeit. Studies in Dire Dawa and South Gondar reported notable contamination, especially in poultry and cattle feed, respectively (Figure 2).

### 3.4 Prevalence of AFB1 in Livestock Feed in Ethiopia

The authors conducted studies on aflatoxin contamination in livestock feed, and the studies covered different regions or study areas within Ethiopia, providing a broad geographic representation. Two main types of study animals were investigated, cattle and poultry, which represented two significant livestock categories commonly exposed to aflatoxin through their feed. The sample types varied, including feed mixes, groundnut, sorghum, maize and others, reflecting the diversity of feed sources examined in the studies. The sample sizes in the studies ranged from relatively small (*n* = 5) to larger samples (*n* = 595). Smaller sample sizes focused on highly localized studies or specific feed types, providing valuable insights into micro‐level contamination. Larger sample sizes offered a broader perspective but often overlooked local nuances. Both approaches were critical for comprehensively understanding AFB1 contamination.

The studies employed various methods for detecting aflatoxin, such as TLC, ELISA and HPLC, and the mean AFB1 levels, measured in micrograms per kilogram (µg/kg), showed variability across studies. One study reported higher levels (715 µg/kg), whereas another reported lower concentrations (8.4 µg/kg). The prevalence rates ranged from 2.4% to 100%, and some of the studies reported very high prevalence rates (100%), indicating a widespread presence of aflatoxin contamination in the samples (Table 1).

**TABLE 1:** The characteristics of the studies in relation to variables of interest extracted from the retrieved works.

Author(s)	Region/Study area	Study animal	Sample type	Sample size	Detection method	Mean AFB1 level (µg/kg)	Prevalence (%)
Fuffa and Urga (2001)	Harari (SNNP)	Cattle	Feed mixes	595	TLC	715	97
Assefa et al. (2012)	Tigray	Cattle	Groundnut	141	ELISA	198.5	96.7
Taye et al. (2016)	East Hararghe	Cattle	Sorghum	90	ELISA	33.1	100
Gizachew et al. (2016)	Addis Ababa (C. Ethiopia)	Cattle	Feed mixes	156	ELISA	213	89.8
Assaye et al. (2016)	West Gojam	Cattle	Maize	30	HPLC	33.5	66.7
Ayelign et al. (2018)	Amhara, Tigray, Oromia, SNNP	Cattle	Feed mixes	126	ELISA	9.4	2.4
Mesfin et al. (2018)	Holetta and Debre Zeit	Cattle	Feed mixes	84	ELISA	8.4	58
Motbaynor et al. (2021)	Dire Dawa	Poultry	Feed mixes	374	ELISA	31.2	81
Kassaw et al. (2022)	Debre Zeit	Poultry	Feed mixes	33	HPLC	70.1	66.7
Fikadu et al. (2022)	Addis Ababa (C. Ethiopia)	Cattle	Feed mixes	90	HPLC	29.2	100
Tefera et al. (2022)	Sidama zone (S. Ethiopia)	Cattle	Feed mixes	240	ELISA	14.9	57.1
Tadele et al. (2023)	South Gondar (NW Ethiopia)	Cattle	Feed mixes	100	ELISA	13.9	96
Mengesha et al. (2023) (a)	Addis Ababa (C. Ethiopia)	Cattle	Feed mixes	5	HPLC	192.8	100
Mengesha et al. (2023) (b)	Addis Ababa (C. Ethiopia)	Poultry	Feed mixes	6	HPLC	74.8	100

Abbreviations: ELISA, enzyme‐linked immunosorbent assay; HPLC, high‐performance liquid chromatography; TLC, thin layer chromatography.

TLC was employed by Fuffa and Urga (2001) in Harari (SNNP). ELISA was the method of choice in studies by Assefa et al. (2012), Taye et al. (2016), Gizachew et al. (2016), Ayelign et al. (2018), Mesfin et al. (2018), Motbaynor et al. (2021) and Tefera et al. (2022). HPLC was used by Assaye et al. (2016), Kassaw et al. (2022), Fikadu et al. (2022), Tadele et al. (2023) and Mengesha et al. (2023) for studies involving both cattle and poultry (Table 1).

**FIGURE 3:**
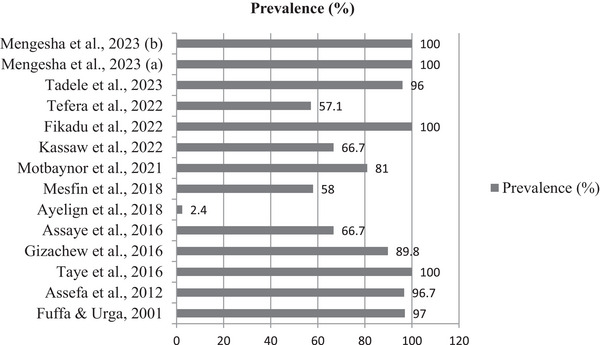
Prevalence of aflatoxin B1 in livestock feed across studies.

The prevalence rates reported by the different authors varied widely across the studies. Prevalence rates ranged from as low as 2.4% to as high as 100% (Figure 3). This wide range indicated significant variability in the extent of aflatoxin contamination in the samples analysed by different authors.

The ANCOVA was conducted to determine whether differences in AFB1 levels were influenced by factors, such as sample size, study animal type, feed type and detection methods. The results indicated that detection methods significantly influenced AFB1 levels (*p* = 0.019), suggesting that different methodologies may yield varying contamination levels. However, no significant differences were observed on the basis of sample size (*p* = 0.796), study animal type (*p* = 0.920) or feed type (*p* = 0.506).

### 3.5 Available Methods of AFB1 Detection in Livestock Feed

**FIGURE 4:**
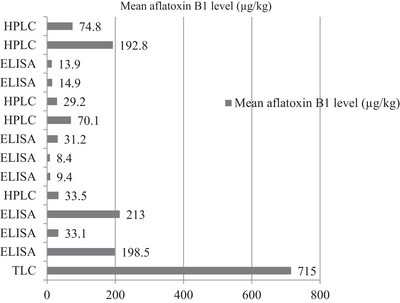
Detection methods and mean aflatoxin B1 levels. ELISA, enzyme‐linked immunosorbent assay; HPLC, high‐performance liquid chromatography; TLC, thin layer chromatography.

The bar graph compared the methods used to detect AFB1 in livestock feed and the corresponding mean aflatoxin levels. The graph included three detection methods: TLC, ELISA and HPLC. The studies reported that ELISA was the most commonly used method, with moderate mean aflatoxin levels across different samples. TLC, used in fewer studies, detected relatively lower mean levels. HPLC, known for its precision and sensitivity, reported the highest mean levels of AFB1, likely reflecting its ability to detect even low concentrations. The findings revealed that the choice of detection method influenced the reported aflatoxin levels. ELISA appeared to be the preferred method due to its accessibility, even though HPLC provided more accurate results (Figure 4).

## 4 Discussion

This systematic appraisal aimed to review the prevalence of AFB1 in livestock feed, particularly in cattle and poultry, as well as the methods of aflatoxin detection in Ethiopia. In this review on the prevalence of aflatoxin and detection methods in the Ethiopian context, the authors reported higher levels of AFB1 in livestock ready‐made feed mixes and groundnut samples. The review presented a range of prevalence rates of AFB1 in livestock feed across different studies, with values ranging from 2.4% (Ayelign et al. 2018) to 100% (Mengesha et al. 2023; Fikadu et al. 2022; Taye et al. 2016). This broad range reflects variability in contamination levels, likely influenced by differences in feed types (e.g., groundnuts are more susceptible to fungal growth), agricultural practices, regional climate conditions conducive to *Aspergillus* growth and storage practices. Identifying the drivers of this variability is essential for designing context‐specific mitigation strategies. The studies that reported very high prevalence rates (100%) indicated a near‐universal presence of aflatoxin contamination in the examined samples. Such findings point out the urgent need for proactive measures to prevent contamination, particularly in high‐risk feed types like groundnuts and feed mixes.

The spatial distribution of the studies revealed varying contamination levels across different regions of Ethiopia. The authors from urban areas like Addis Ababa (Mengesha et al. 2023; Fikadu et al. 2022) and districts like Dire Dawa (Motbaynor et al. 2021) and South Gondar (Tadele et al. 2023) reported notable contamination, emphasizing the importance of understanding regional variations. Urban areas may face greater challenges due to concentrated livestock farming, increased reliance on commercially prepared feed and suboptimal storage conditions. Conversely, rural areas may experience contamination stemming from poor post‐harvest practices and limited awareness of feed safety protocols. This insight is critical, as aflatoxin contamination can be influenced by local environmental factors, such as temperature and humidity, which are conducive to fungal growth and mycotoxin production. These regional differences demonstrate the need for targeted interventions and policies that consider the unique challenges faced by each area to ensure more effective management of aflatoxin contamination (Kwigizile et al. 2024; Neogi et al. 2024; Stutt et al. 2023; Tueller et al. 2023). Policy interventions should prioritize capacity building for farmers and feed producers, particularly in regions with the highest contamination levels, while fostering collaboration between local authorities and international stakeholders.

The sample sizes in the studies ranged from a small number (*n* = 5) (Mengesha et al. 2023) to larger samples (*n* = 595) (Fuffa and Urga 2001). The sample size does influence the precision and generalizability of prevalence estimates, with smaller sample sizes being more prone to variability (Lin 2018). Larger sample sizes typically provide more reliable and accurate estimates of contamination prevalence, which is crucial when formulating national guidelines for aflatoxin management. However, smaller studies may offer valuable insights into localized contamination patterns and specific risk factors that larger studies might overlook. Balancing both perspectives is essential for a comprehensive understanding of the issue.

The systematic review provided an overview of the diverse methods employed for aflatoxin detection in Ethiopian livestock feed. The use of TLC was pointed out by Fuffa and Urga (2001), whereas ELISA was employed in the studies conducted by Assefa et al. (2012), Gizachew et al. (2016), Ayelign et al. (2018) and Mesfin et al. (2018). HPLC was noted as a detection method in studies by Assaye et al. (2016), Kassaw et al. (2022), Mengesha et al. (2023) and Fikadu et al. (2022). Although these methods provide reliable results, the need for skilled personnel and laboratory infrastructure limits their accessibility, particularly in rural areas. Recent advancements in rapid detection techniques, such as PCR and near‐infrared hyperspectral imaging (HSI), are emerging as promising alternatives for the quick and effective detection of aflatoxins in agricultural products (Yilma et al. 2019; Narayanan and Reddy 2023). These technologies, if adopted, could revolutionize feed safety monitoring in Ethiopia by enabling faster and more accurate detection at lower costs, but their implementation requires significant investment in training and infrastructure.

The mean AFB1 levels in µg/kg varied widely across studies, and some of the studies reported relatively low levels, 8.4 and 9.4 µg/kg, as reported by Ayelign et al. (2018) and Mesfin et al. (2018), respectively. In contrast, other studies reported higher concentrations (Gizachew et al. 2016; Mengesha et al. 2023; Fuffa and Urga 2001; Assefa et al. 2012). This variability could have been influenced by factors, such as local agricultural practices, crop types and the presence of aflatoxin‐producing fungi. Additionally, the differences in AFB1 levels may also be related to variations in feed ingredients and the specific fungal species present in the samples. Such findings demonstrate the importance of addressing contamination at every stage of the feed supply chain, from pre‐harvest practices to storage and transportation. Integrating GAP and HACCP systems can significantly mitigate these risks.

The EU has established maximum limits for AFB1 in various food and feed categories (Verstraete 2008), and the Codex Alimentarius Commission and the US FDA also provide guidelines (Meneely et al. 2023). The EU has set AFB1 limits at 5 µg/kg for feed materials and compound feed for dairy animals (Girolami et al. 2022). These international standards are essential for ensuring the safety of animal feed and minimizing the risk of aflatoxin exposure in the food chain. For certain foodstuffs intended for direct human consumption, the limit is even lower. If the mean AFB1 levels exceed the established limits, it suggests a potential risk to animal and human health (Al‐Jaal et al. 2019; Panel et al. 2020). The regulatory framework of Ethiopia must align more closely with these international benchmarks to ensure market competitiveness and safeguard public health. Tailored regulations that account for the local context, combined with stronger enforcement mechanisms, are critical for achieving this goal. Different livestock feed types may have distinct regulatory limits, and the limits for AFB1 in poultry feed might differ from those for cattle feed. Therefore, it is crucial to consider the type of livestock feed and the specific regulations for each type when comparing with international standards. The type of livestock feed should be taken into account when comparing with international standards (Dinkissa and Hailu 2022; Kassaw et al. 2022; Coppock et al. 2018).

## 5 Conclusion

This systematic review demonstrates the widespread presence and significant risks of AFB1 contamination in livestock feed across Ethiopia, with prevalence rates ranging from 2.4% to 100%. Urban areas and regions like Addis Ababa, Dire Dawa and South Gondar reported notably higher contamination levels, revealing the influence of localized agricultural practices, storage conditions and climatic factors. The variability in contamination levels across studies emphasizes the need for region‐specific interventions, informed by rigorous monitoring and tailored mitigation strategies.

The analysis revealed gaps in detection capacities, with reliance on traditional methods such as TLC and ELISA, whereas advanced techniques like HPLC remain underutilized due to resource constraints. Promising alternatives like PCR and HSI offer opportunities to improve detection accuracy and timeliness but require investment in infrastructure and training.

The regulatory framework of Ethiopia must align more closely with international standards, such as those of the Codex Alimentarius and the EU, to ensure feed safety and mitigate health risks to animals and humans. This alignment should include setting enforceable limits for AFB1 in livestock feed and strengthening enforcement mechanisms. Enhanced storage practices, the adoption of GAP and comprehensive training programmes for farmers and feed producers are crucial to reducing contamination risks.

By implementing these measures and fostering collaboration among stakeholders, Ethiopia can effectively address the challenges posed by AFB1 contamination. This approach not only safeguards animal and public health but also strengthens the livestock sector and enhances food security.

## Author Contributions

Sisay Weldegebriel Zeweld wrote the original draft, conceptualized and developed the methodology and contributed to the review and editing. Enquebaher Kassaye Tarekegn reviewed and edited the draft, and Meressa Abraha Welearegay also reviewed and edited the manuscript. All authors have read and approved the final manuscript.

## Ethics Statement

The authors have nothing to report.

## Conflicts of Interest

The authors declare no conflicts of interest.

### Peer Review

The peer review history for this article is available at https://www.webofscience.com/api/gateway/wos/peer‐review/10.1002/vms3.70405.

## Data Availability

The authors have nothing to report.

## References

[vms370405-bib-0001] Alameri, M. M. , A. S. Kong , M. N. Aljaafari , et al. 2023. “Aflatoxin Contamination: An Overview on Health Issues, Detection and Management Strategies.” Toxins (Basel) 15, no. 4: 246. 37104184 10.3390/toxins15040246 PMC10140874

[vms370405-bib-0002] Al‐Jaal, B. A. , M. Jaganjac , A. Barcaru , P. Horvatovich , and A Latiff . 2019. “Aflatoxin, Fumonisin, Ochratoxin, Zearalenone and Deoxynivalenol Biomarkers in Human Biological Fluids: A Systematic Literature Review, 2001–2018.” Food and Chemical Toxicology 129: 211–228. 31034935 10.1016/j.fct.2019.04.047

[vms370405-bib-0003] Assaye, M. , N. Gemeda , and G Weledesemayat . 2016. “Aspergillus Species and Aflatoxin Contamination of Pre and Post‐Harvest Maize Grain in West Gojam, Ethiopia.” Journal of Food Science and Nutrtion 2, no. 2: 1–7.

[vms370405-bib-0004] Assefa, D. , M. Teare , and H Skinnes . 2012. “Natural Occurrence of Toxigenic Fungi Species and Aflatoxin in Freshly Harvested Groundnut Kernels in Tigray, Northern Ethiopia.” Journal of the Drylands 5, no. 1: 377–384.

[vms370405-bib-0005] Ayelign, A. , A. Z. Woldegiorgis , A. Adish , and S De Saeger . 2018. “Total Aflatoxins in Complementary Foods Produced at Community Levels Using Locally Available Ingredients in Ethiopia.” Food Additives & Contaminants: Part B 11, no. 2: 111–118. 10.1080/19393210.2018.1437784 29421965

[vms370405-bib-0006] Berrang‐Ford, L. , T. Pearce , and J. D Ford . 2015. “Systematic Review Approaches for Climate Change Adaptation Research.” Regional Environmental Change 15: 755–769.

[vms370405-bib-0007] Coppock, R. W. , R. G. Christian , and B. J Jacobsen . 2018. “Aflatoxins.” In Veterinary Toxicology, 983–994. Elsevier.

[vms370405-bib-0008] Desta, A. G. 2023. “Nutritional Content Analysis of Crop Residues in Three Agroecologies in East Gojjam Zone.” Scientific World Journal 2023: 1974081. 37214644 10.1155/2023/1974081 PMC10199800

[vms370405-bib-0009] Dinkissa, A. F. , and Y Hailu . 2022. “Effect of Aflatoxin Contamination in Dairy Products and Its Toxicity on Public Health: The Case of Ethiopian Dairy Sector: A Review.” Diyala Agricultural Sciences Journal 14, no. 2: 73–86.

[vms370405-bib-0010] Ebrahimi, S. H. 2020. “Feeding Complete Concentrate Pellets Containing Ground Grains or Blend of Steam‐Flaked Grains and Other Concentrate Ingredients in Ruminant Nutrition—A Review.” Annals of Animal Science 20, no. 1: 11–28.

[vms370405-bib-0011] Fikadu, J. , B. Tamir , U. Galmessa , and K Effa . 2022. “Feed Quality, Prevalence of Aflatoxin Contamination in Dairy Feed and Raw Milk in Oromia Special Zone Surrounding Finfinne, Ethiopia.” Asian Journal of Dairy and Food Research 41, no. 1: 8–14.

[vms370405-bib-0012] Fuffa, H. , and K Urga . 2001. “Survey of Aflatoxin Contamination in Ethiopia.” Ethiopian Journal of Health Sciences 11, no. 1: 17–25.

[vms370405-bib-0013] Garcia‐Alvarez‐Coque, J. M. , I. Taghouti , and V. Martinez‐Gomez . 2020. “Changes in Aflatoxin Standards: Implications for EU Border Controls of Nut Imports.” Applied Economic Perspectives and Policy 42, no. 3: 524–541.

[vms370405-bib-0014] Getabalew, M. , T. Alemneh , and D Akeberegn . 2019. “Dairy Production in Ethiopia‐Existing Scenario and Constraints.” Biomedical Journal of Scientific & Technical Research 16, no. 5: 12304–12309.

[vms370405-bib-0015] Girolami, F. , A. Barbarossa , P. Badino , et al. 2022. “Effects of Turmeric Powder on Aflatoxin M1 and Aflatoxicol Excretion in Milk From Dairy Cows Exposed to Aflatoxin B1 at the EU Maximum Tolerable Levels.” Toxins (Basel) 14, no. 7: 430. 35878168 10.3390/toxins14070430 PMC9317782

[vms370405-bib-0016] Gizachew, D. , B. Szonyi , A. Tegegne , J. Hanson , and D Grace . 2016. “Aflatoxin Contamination of Milk and Dairy Feeds in the Greater Addis Ababa Milk Shed, Ethiopia.” Food Control 59: 773–779.

[vms370405-bib-0017] Karangiya, V. , H. Savsani , and N Ribadiya . 2016. “Use of Densified Complete Feed Blocks as Ruminant Feed for Sustainable Livestock Production: A Review.” Agricultural Reviews 37, no. 2: 141–147.

[vms370405-bib-0018] Kassaw, T. S. , Y. C. Megerssa , and F. T Woldemariyam . 2022. “Occurrence of Aflatoxins in Poultry Feed in Selected Chicken Rearing Villages of Bishoftu Ethiopia.” Veterinary Medicine: Research and Reports 13: 277–286. 10.2147/VMRR.S384148 PMC9586162 36277466

[vms370405-bib-0019] Kemboi, D. C. , G. Antonissen , P. E. Ochieng , et al. 2020. “A Review of the Impact of Mycotoxins on Dairy Cattle Health: Challenges for Food Safety and Dairy Production in Sub‐Saharan Africa.” Toxins (Basel) 12, no. 4: 222. 32252249 10.3390/toxins12040222 PMC7232242

[vms370405-bib-0020] Kitigwa, S. 2023. Occurrence of Aflatoxins and Associated Risk Factors in Dairy Value Chain in Selected Districts of Three Agro‐Ecological Zones in Tanzania . NM‐AIST.

[vms370405-bib-0021] Kwigizile, O. H. , E. R. Mbega , A. Mushongi , and M Philipo . 2024. “Prevalence of Aflatoxigenic Fungi and Contamination in Soils and Maize Grains From Aflatoxin‐Hot Spot Areas in Tanzania.” Journal of Food Composition and Analysis 135: 106608.

[vms370405-bib-0022] Lin, L. 2018. “Bias Caused by Sampling Error in Meta‐Analysis With Small Sample Sizes.” PLoS ONE 13, no. 9: e0204056. 30212588 10.1371/journal.pone.0204056 PMC6136825

[vms370405-bib-0023] Mahato, D. K. , K. E. Lee , M. Kamle , et al. 2019. “Aflatoxins in Food and Feed: An Overview on Prevalence, Detection and Control Strategies.” Frontiers in Microbiology 10: 2266. 31636616 10.3389/fmicb.2019.02266 PMC6787635

[vms370405-bib-0024] McCuistion, K. C. , P. H. Selle , S. Y. Liu , and R. D Goodband . 2019. “Sorghum as a Feed Grain for Animal Production.” In Sorghum and Millets, 355–391. AACC International.

[vms370405-bib-0025] Meneely, J. P. , O. Kolawole , S. A. Haughey , S. J. Miller , R. Krska , and C. T Elliott . 2023. “The Challenge of Global Aflatoxins Legislation With a Focus on Peanuts and Peanut Products: A Systematic Review.” Exposure and Health 15, no. 2: 467–487.

[vms370405-bib-0026] Mengesha, G. , T. Bekele , H. Ashagrie , and A. Z Woldegiorgis . 2023. “Level of Aflatoxins in Dairy Feeds, Poultry Feeds, and Feed Ingredients Produced by Feed Factories in Addis Ababa, Ethiopia.” Mycotoxin Research 40, no. 2: 309–318. 10.1007/s12550-024-00531-8 38530632

[vms370405-bib-0027] Mesfin, R. , G. Assefa , and F Assefa . 2018. “Determination of Aflatoxin in Dairy Feeds and Milk in Some Selected Areas of Ethiopia.” Food and Environment Safety 17, no. 3: 286–299.

[vms370405-bib-0028] Motbaynor, A. , D. Kassaye , M. Keffale , and P Wasihun . 2021. “Magnitude of Aflatoxigenic *Aspergillus* Species, Level of Aflatoxin B1, and Associated Factors in Stored Feed at Poultry Farms in Dire Dawa, Ethiopia.” Veterinary Medicine International 2021: 6638083. 34721834 10.1155/2021/6638083 PMC8553476

[vms370405-bib-0029] Narayanan, R. , and M. K Reddy . 2023. “Techniques Used for Detection of Aflatoxins in Milk: A Review.” Agricultural Reviews 44, no. 1: 39–45.

[vms370405-bib-0030] Ndudzo, A. , J. Pullen , T. Magwaba , et al. 2023. “Incorporation of Functional Feed Ingredients to Substitute Antimicrobials in Animal Nutrition: Opportunities for Livestock Production in Developing Countries.” International Journal of Livestock Production 14, no. 2: 44–57..

[vms370405-bib-0031] Negash, D. 2018. “A Review of Aflatoxin: Occurrence, Prevention, and Gaps in Both Food and Feed Safety.” Journal of Applied Microbiological Research 1, no. 1: 35–43.

[vms370405-bib-0032] Neogi, S. B. , M. N. Uddin , M. Arif , et al. 2024. “Influence of Climatic Factors on Spatio‐Temporal Variabilities in the Occurrence of Aflatoxins in Major Food Grains Available at Retail Shops in Bangladesh.” Asian‐Australasian Journal of Food Safety and Security 8, no. 2: 32–47.

[vms370405-bib-0033] EFSA Panel on Contaminants in the Food Chain (CONTAM) . Schrenk, D. , M. Bignami , et al. 2020. “Risk Assessment of Aflatoxins in Food.” EFSA Journal 18, no. 3: e06040. 32874256 10.2903/j.efsa.2020.6040 PMC7447885

[vms370405-bib-0034] Sangvikar, R. V. 2021. “Food Safety Concern Related to Aflatoxins and Control.” Fungi Bio‐Prospects in Sustainable Agriculture, Environment and Nano‐technology 3: 347–380.

[vms370405-bib-0035] Setsetse, K. 2019. The Impact of Storage Facilities on Animal Feed Quality With Reference to Mycotoxin Contamination Around Ngaka Modiri Molema District, North West Province: North‐West University (South Africa).

[vms370405-bib-0036] Shiferaw, F. , B. Asmare , M. H. Melekot , and W. F Pellikaan . 2022. “Dried‐Atella as an Affordable Supplementary Feed Resource for a Better Sheep Production: In the Case of Washera Lambs in Ethiopia.” Translational Animal Science 6, no. 4: txac146. 36479382 10.1093/tas/txac146 PMC9721381

[vms370405-bib-0037] Stutt, R. O. , M. D. Castle , P. Markwell , R. Baker , and C. A Gilligan . 2023. “An Integrated Model for Pre‐and Post‐Harvest Aflatoxin Contamination in Maize.” Npj Science of Food 7, no. 1: 60. 37980424 10.1038/s41538-023-00238-7 PMC10657429

[vms370405-bib-0038] Tadele, F. , B. Demissie , A. Amsalu , et al. 2023. “Aflatoxin Contamination of Animal Feeds and Its Predictors Among Dairy Farms in Northwest Ethiopia: One Health Approach Implications.” Frontiers in Veterinary Science 10: 1123573. 37035821 10.3389/fvets.2023.1123573 PMC10076730

[vms370405-bib-0039] Tadesse, A. , M. Urge , and M Girma . 2020. “Effects of Replacing Concentrates With Atella (a Byproduct of Local Beer) on Growth Performance of Hararghe Highland Sheep in Ethiopia.” Livestock Research Rural Development 32, no. 7: 109.

[vms370405-bib-0040] Taye, W. , A. Ayalew , A. Chala , and M Dejene . 2016. “Aflatoxin B1 and Total Fumonisin Contamination and Their Producing Fungi in Fresh and Stored Sorghum Grain in East Hararghe, Ethiopia.” Food Additives & Contaminants: Part B 9, no. 4: 237–245. 10.1080/19393210.2016.1184190 27161169

[vms370405-bib-0041] Tefera, W. , G. E. Vegarud , M. Taye , and T Taye . 2022. “Aflatoxin Contamination in Dairy Feed During Wet and Dry Seasons in Selected Rural Areas of Sidama Zone in Southern Ethiopia.” International Journal of Agricultural Science and Food Technology 8, no. 1: 078–082.

[vms370405-bib-0042] Tueller, G. , R. Kerry , and S. G Young . 2023. “Spatial Investigation of the Links Between Aflatoxins Legislation, Climate, and Liver Cancer at the Global Scale.” Spatial and Spatio‐Temporal Epidemiology 46: 100592. 37500231 10.1016/j.sste.2023.100592

[vms370405-bib-0043] Verstraete, F. 2008. “European Union Legislation on Mycotoxins in Food and Feed: Overview of the Decision‐Making Process and Recent and Future Developments.” In Mycotoxins: Detection Methods, Management, Public Health and Agricultural Trade, 77–99. CABI Publishing.

[vms370405-bib-0044] Yilma, S. , K. Sadessa , and D Kebede . 2019. “Fungal Infections and Aflatoxin Contamination in Maize Grains Collected From West Showa and East Wallega Zones, Ethiopia.” International Journal of Current Research and Review 11: 16–22.

[vms370405-bib-0045] Zewdie, W. , M. Yoseph , and B Wouters . 2011. “Peri‐Urban Dairy Production System in Relation With Feed Availability in the Highlands of Ethiopia.” World Applied Sciences Journal 13, no. 7: 1712–1719.

